# Does the neighborhood built environment moderate the effectiveness of a weight-loss intervention for mothers with overweight or obesity? Findings from the Healthy Eating and Active Living Taught at Home (HEALTH) study

**DOI:** 10.1186/s12966-022-01368-z

**Published:** 2022-10-01

**Authors:** Amanda S. Gilbert, Deborah Salvo, Rachel G. Tabak, Debra Haire-Joshu

**Affiliations:** 1 Prevention Research Center in St. Louis, Brown School at Washington University in St. Louis, St. Louis, MO USA; 2 Center for Diabetes Translation Research, Washington University in St. Louis, St. Louis, MO USA; 3 Department of Kinesiology and Health Education, College of Education, The University of Texas at Austin, Bellmont Hall (BEL) at 2109 San Jacinto Blvd., Austin, TX 78712 USA

**Keywords:** Obesity, Built Environment, Health behaviors, Lifestyle intervention, Maternal

## Abstract

**Background:**

Women of childbearing age are vulnerable to weight gain and experience a high prevalence of obesity due to pregnancy and stressors of parenthood. Lifestyle interventions such as the Healthy Eating and Active Living Taught at Home (HEALTH) study have been effective for weight loss; however, little is known about how the built environment (parks, transit, grocery stores, fast food, walkability etc.), where participants live might modify intervention effectiveness. This study examined whether characteristics of the neighborhood built environment modified effectiveness of the HEALTH study on weight loss.

**Methods:**

Secondary data analysis was conducted using data from HEALTH. Using GIS, buffers were built around participant addresses to capture distance to and availability of food (grocery store, convenience store, fast food) and urban design and transit (parks, street connectivity, transit) built environment characteristics. Built environment characteristics were dichotomized into low and high density and distance. Likelihood ratio tests for interaction were conducted to determine if built environment characteristics modified intervention effectiveness on Body mass index (BMI) and waist circumference (WC). Mixed effects linear regression models were then run to estimate the effect of the HEALTH intervention on weight outcomes at 24-months across both strata of built environment characteristics.

**Results:**

The analytic sample (*n* = 151) had baseline mean BMI 34.9 (SD = 5.8) and mean WC 46.0 cm (SD4.9). All urban design and transit and all food environment characteristics modified HEALTH effectiveness on one or both weight outcomes. The built environment modified the HEALTH intervention such that it was mostly effective for mothers residing in neighborhoods with low transit access, low street connectivity, high park access, and low access to grocery stores, convenience stores, and fast food.

**Conclusions:**

Result show the HEALTH was most effective for women residing neighborhoods with built environment characteristics suggestive of suburban neighborhood typology. To maximize impact for mothers residing in all types of neighborhoods, future research should explore scaling up HEALTH in suburban settings, while adapting HEALTH to maximize effectiveness in compact neighborhoods most likely, urban core neighborhoods.

**Supplementary Information:**

The online version contains supplementary material available at 10.1186/s12966-022-01368-z.

## Background

Obesity is an urgent public health problem. An estimated 13% of the global population and 42% of the US adult population have obesity, putting them at higher risk for diabetes, heart disease, hypertension, cancer, Alzheimer’s, psychological conditions, physical impairment, and premature death [1–7]. Although extensive work has been conducted in understanding the determinants and designing interventions for weight-loss among various sub-population groups, including children and adolescents, older adults, and more recently among some racial and ethnic minorities, less research is available on how to prevent and treat obesity among women of childbearing age (20 to 39 years). It has been documented that during this stage in life women experience an increased vulnerability to weight gain and the development of obesity [8, 9]. Indeed, among women, the prevalence of obesity increases from 20–24% during adolescence (12–19 years old), to 33–57% during the childbearing years [8]. During the childbearing years, women experience an average weight gain per year of one to two pounds [9]. It has also been reported that approximately 23% of women gain an excess of 44 pounds or more above their healthy weight range upper limit between the ages of 18 and 55 years old [8, 9].

One aspect that makes women in this stage of life particularly vulnerable to weight gain is pregnancy, as well as the stressors associated with parenthood. Excessive weight gain during pregnancy and post-partum weight retention can make it difficult for women to return to a healthy weight [10–15]. Additionally, early adulthood is a period of life in which many transitions take place (new jobs, marriage, parenthood), increasing responsibilities and roles. During this period, many women have to balance demands from work and childcare, experiencing time constraints and increased financial costs, limiting their ability to maintain healthy lifestyles [8, 16, 17]. Beyond being a concern for women’s health [1–5, 7, 18, 19], weight gain among mothers has been shown to increase the likelihood that offspring will develop overweight and obesity later in life [20–23]. This intergenerational transmission of obesity is expected to continue to accelerate the obesity epidemic resulting in worsening health outcomes across generations [21, 24].

One way in which weight-loss or weight-gain prevention interventions have been developed and tested to reach women of childbearing age is through home visiting programs. Parents as Teachers (PAT) is a national home visiting organization providing free home visits for families with high needs (i.e., low educational attainment, low income, a parent or child with disabilities/chronic health condition, recent immigrant, parent with mental illness, or unstable housing). The goal of PAT is to promote child development and health through parent education and connection to resources [25]. Recently, PAT partnered with the research team at Washington University to implement the Healthy Eating & Active Living Taught at Home (HEALTH) study [26].The HEALTH study tested a 24-month lifestyle intervention on improving weight outcomes among mothers with obesity, through a home-delivered adaptation of the Diabetes Prevention Program [26]. When compared to usual care, the intervention group was significantly more likely to achieve and maintain a 5% weight loss at 24 months and show improvements at 12 and 24 months in waist circumference, blood pressure, and behavioral outcomes, defined by eating patterns and physical activity [26].

The socio-ecological model of health behaviors underscores the influence of environmental factors on individual-level health behaviors, and the interaction across levels of influence (including, for instance, interactions between the inter-personal level, at which interventions like HEALTH take place, and the environmental level) [27, 28]. The built environment includes all aspects of a person’s environment which are human-made or modified, including buildings, spaces and urban design and infrastructure elements [29]. Understanding if the neighborhood built environment plays a moderating role for the effectiveness of the intervention could provide important insights for program tailoring and scale-up. Other studies have reported significant differences in mean BMI and waist circumference by neighborhood built environment characteristics such as access to parks, transit, grocery stores, fast food restaurants, convenience stores, and by walkability scores [30–37]. However, few studies have examined if and how the effectiveness of lifestyle-modification interventions, such as the one tested in the HEALTH study, varies depending on the characteristics of the built environment of neighborhoods where participants live. Among the available studies examining moderation of intervention effectiveness by built environment features, most have focused on either physical activity or nutrition outcomes [38–44], with fewer studies assessing moderating effects on obesity or weight-related outcomes [45, 46]. Further, almost none of these studies are specific to women of childbearing age. Using an ecological approach, the aim of this study was to examine whether different characteristics of the neighborhood built environment modified the effectiveness of the HEALTH intervention on weight loss outcomes (reductions in BMI and waist circumference).

## Methods

### Study design and parent study

We conducted a secondary analysis using HEALTH study data. Briefly, the HEALTH study aimed to improve weight outcomes for mothers of preschool children (ages 3–5) and took place between 2012 and 2016. Additional inclusion criteria for the mother included having obesity and residing in the St. Louis, Missouri region. Families that were already enrolled in the PAT program were recruited via a flyer at events, through providers, at childcare, or mailed. Families interested in participating were asked to contact the research team. Families were randomized to usual care (PAT program) or intervention (PAT + HEALTH intensive lifestyle intervention), which consisted of education and counseling aimed at physical activity and dietary behavior change [26]. The HEALTH intensive lifestyle intervention was modeled after the Diabetes Prevention Program and included strategies targeting intrapersonal (e.g., self-monitoring), interpersonal (e.g., family meal time), and home environment (e.g., food access at home) factors related to health behavior change. Behavioral change strategies used in the intervention included goal setting (e.g., set goal to reduce inactive time), nutritional and physical activity education (e.g., learn the benefits of eating a healthy breakfast), stimulus control (e.g., search home for problem food cues and make changes), and skill development (e.g., read food labels). Extensive information about the parent study, including details about the behavior change strategies implemented, has been published elsewhere (see Additional file 1 Appendix Table 1of cited article by Haire-Joshu and colleagues for full description of the behavior change strategies included in the intervention) [26]. Our sample for this secondary analysis examining the moderating effects of neighborhood built environment characteristics on the HEALTH intervention consisted of all HEALTH study participants with complete outcome data at baseline and 24 months, and whose addresses were available for geocoding. In the parent study, 23% of participants for which data were collected at baseline moved or could not be located at follow-up. These participants did not complete the 24-month follow-up, and as such, were not included in the analysis reported in this paper, which includes only participants that resided in the same location throughout the study period.

**Table 1:** Baseline characteristics of the final analytic sample (HEALTH study, 2012–2016)^a^

Participants	Total	Usual care	Intervention	*p*-value
	(*n* = 151)	(*n* = 83)	(*n* = 68)	
BMI^b^, M (SD)	34.86 (5.76)	35.66 (5.67)	33.89 (5.77)	0.06
Waist circumference, cm, M (SD)	46.02 (4.89)	46.74 (4.75)	45.15 (4.95)	0.05
Age, years M (SD)	32.96 (5.49)	33.04 (5.39)	32.86 (5.63)	0.85
Race, n (%)				0.26
Black or African American	44 (29.14)	25 (30.12)	19 (27.94)	
White	93 (61.59)	51 (61.45)	42 (61.76)	
Other	11 (7.28)	7 (8.43)	4 (5.88)	
Unknown, not reporting race	3 (1.99)	0 (0.00)	3 (4.41)	
College or more, n (%)	131 (86.75)	75 (90.36)	56 (82.35)	0.23
Household annual income, n (%)				0.88
< 30,000	49 (34.03)	29 (35.37)	20 (32.26)	
30,000–74,999	57 (39.58)	31 (37.80)	26 (41.94)	
≥ 75,000	38 (26.39)	22 (26.83)	16 (25.81)	
Presently married	94 (62.25)	51 (61.45)	43 (63.24)	0.95

^a^Based on final analytic sample (*n* = 151)

^b^Body mass index

## Measures

### Outcome measures

Height, weight, and waist circumference were objectively measured following standard procedures from the National Health and Nutrition Examination Survey [47]. Height and weight were used to obtain BMI using the standard formula [47]. For this analysis, BMI and waist circumference are treated as continuous variables.

### Built environment measures

**Fig. 1:**
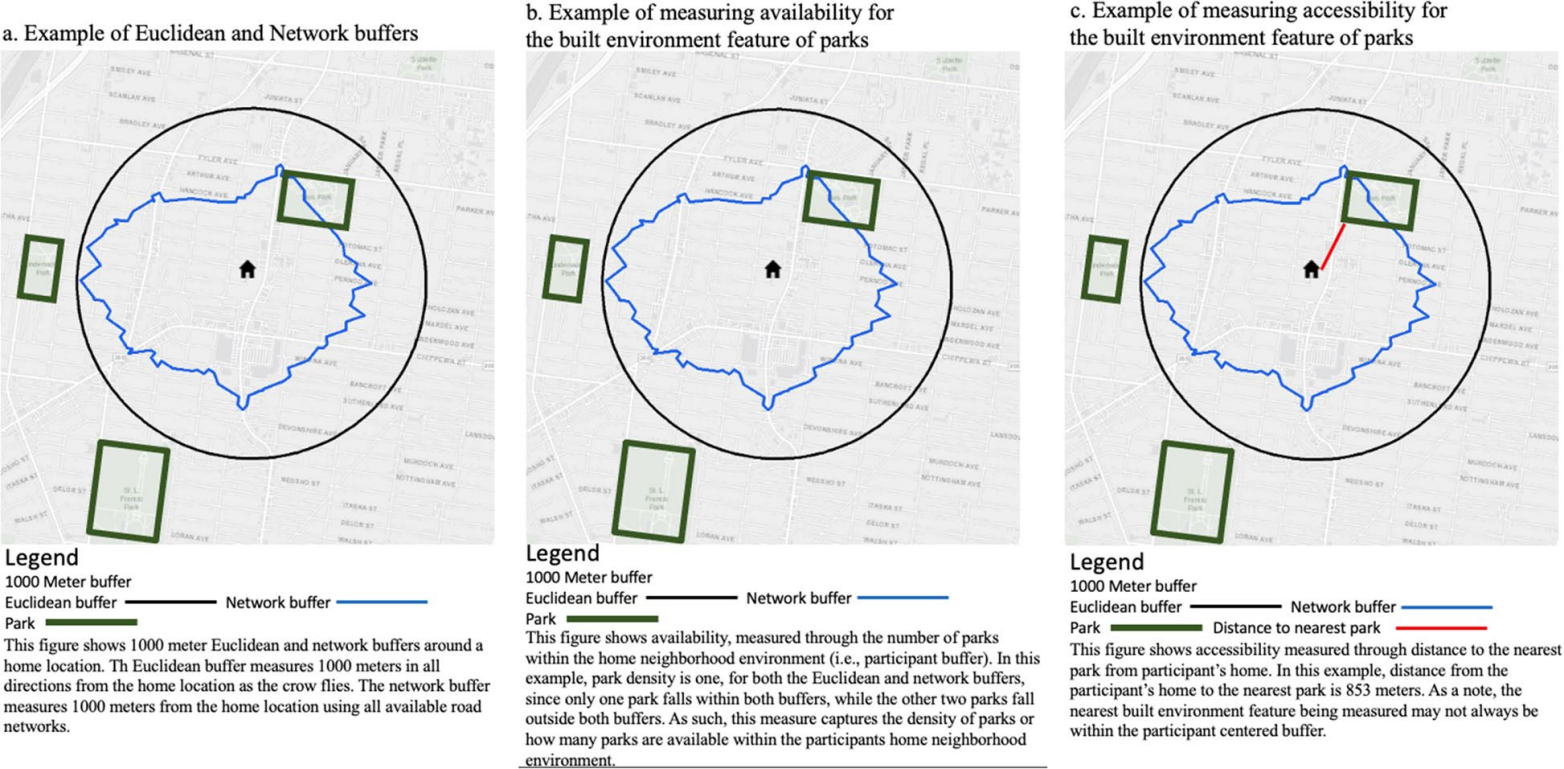
**a **Example of Euclidean and network buffers. This figure shows 1000 meter Euclidean and network buffers around a home location. The Euclidean buffer measures 1000 meters in all directions from the home location as the crow flies. The network buffer measures 1000 meters from the home location using all available road networks. **b** Examples of measuring availability for the built environment feature of parks. This figure shows availability, measured through the number of parks within the home neighborhood built environment (i.e. participant buffer). In this example, park density is one, for both the Euclidean and network buffers, since only Park 1 falls within both buffers, while Park 2 falls outside both buffers. As such, this measure captures the density of parks or how many parks are available within the participants home neighborhood environment. **c** Examples of measuring accessibility for the built environment feature of parks. This figure shows accessibility measured through distance to the par from participant's home. In this example, distance from the particpant's home to the nearest park (Park 1) is 853 meters. As a note, the nearest built environment feature being measured, may not always be within the participant centered buffer

After geocoding all participant addresses, we built a series of participant centric buffers of varying radii (250, 500, 100, 1500) in meters. Given that adults walk at an average speed of 5 km per hour, these buffer radii represent approximate walking times ranging from 2 to 20 min [48]. Two types of buffers were used, including Euclidian buffers, measured as the crow flies, as well as network buffers, measured through underlying road networks to find all possible routes from the participant to the end point (Fig. 1a). In addition to buffer-based variables, some distance-based variables were calculated using the road network. We chose buffer-based and distance-based variables to represent two different dimensions of geospatial access. Buffer-based variables measure *availability* (i.e., diversity and number of options) of a built environment feature within the home neighborhood (i.e., buffer) of a participant (Fig. 1b). For example, how many different options does a participant have in their neighborhood built environment for food? The distance-based variables measure *accessibility (i.e., distance traveled along road networks)* to the nearest built environment feature from the participants home (Fig. 1c). For example, how easy is it for a participant to reach the nearest park to their home?

#### Buffer-based variables

We built a series of buffer-based indicators at each of the buffer types (Euclidian, network) and sizes (250, 500, 1000, 1500 m). Next, we conducted a sensitivity analysis to determine for each built environment buffer-based measure, which specific buffer (type and size) was most strongly correlated with the outcomes of interest (see Additional file 1. Appendix Sect. 1). Based on this analysis, number of parks, Metrobus stops, Metrolink stops, all-transit stops, fast food locations, convenience stores, and grocery stores within each buffer were computed. Street connectivity was measured as counts of three-or-more-way street intersections within each buffer. This is the most frequently used indicator for assessing street connectivity in the US and in international studies, since restricting the indicator to include only 4-or-more-way intersections would result in reduced environmental variability due to the amount of urban sprawl typical of US cities [49]. Finally, population density was estimated using census block group total population size data and intersecting it with each participant's buffer. Through spatial apportionment, the slices of each census block group intersecting the buffer were used to compute a weighted average, to estimate total population size for each participant-centric buffer. This number was divided by the buffer area to estimate population density per buffer and expressed as total population per Kilometer squared.

#### Distance-based variables

We measured distance-based variables by calculating the nearest route in meters from participant address to nearest built environment feature using the road network system. These distance-based variables included distances along the road network to the nearest grocery store, park, transit stop of any kind, fast food location, and convenience store.

For analyses, all buffer-based and distance-based variables were dichotomized. Buffer-based variables of parks, convenience stores, grocery stores, and Metrolink stops were dichotomized as less than one or greater than or equal to one. For buffer-based variables with higher counts the median was used. Fast food was dichotomized at the median of four, and three-or-more-way intersections, all transit stops and Metrobus stops were dichotomized at the median of 21. All distance-based variables were dichotomized at the median, which ranged from a low of 742 m to a high of 1,344 m. It was decided to dichotomize all built environment variables to allow for easier interpretation. The main goal of our work with this analysis was to answer the question of whether, for example: “does the HEALTH intervention work better for people that have access to more parks in their neighborhood than for those who have less parks?”. Hence, built environment variable dichotomization allowed for splicing the modeling results accordingly, across the two levels of the moderating variable. Future work focused on examining the shape of the associations should consider using continuous variables. All spatial measures were built using ArcGIS 10.6 (ESRI Inc).

All of the raw spatial data used to build indicators of the food and built environment were sourced from Environmental Systems Research Institute (ESRI)’s data package, which in itself includes data from both public (e.g., US Census, US Department of Transportation, State and Local Departments of Parks and Recreation, etc.), and proprietary data sources (e.g., ESRI Business Analyst – from which all our food environment data were sourced).

### Statistical analysis

We conducted descriptive analyses by computing counts and percentages for outcomes of interest and built environment measures. For all outcome variables, normality was assessed using skewness and kurtosis statistics, as well as visual assessments of histograms. Since both variables were found to be normally distributed, no data transformations were necessary. Next, we ran a series of likelihood ratio tests for interaction to determine if built environment variables modified the effect of the intervention on BMI and/or on waist circumference. We considered any p-value lower than 0.15 as evidence of potential effect measure modification, warranting further exploration of stratum specific estimates [50]. Mixed effects linear regression models, accounting for the clustering effect of study sites, were run to estimate the effect of the HEALTH intervention, relative to the usual care arm, on the outcomes at 24-months (while adjusting for the outcome at baseline) [51, 52] across both strata of the given built environment variable. Of the 179 participants in the parent study, 156 completed the 24-month follow up assessment, and among them, 151 provided valid residential addresses for geocoding. The analytic sample did not significantly differ from the full parent study sample in terms of sociodemographic characteristics, except for income, which was significantly higher among the analytic sample. Further, income as well as population density, are often highly correlated with built environment characteristics [53–56]. Because of this, we ran unadjusted, density-adjusted, and income-adjusted models. All statistical analyses were conducted using SAS version 9.5 (SAS Institute Inc).

## Results

### Demographics

The analytic sample (*n* = 151, usual care = 83, intervention = 68) included participants with a mean age of 33.0 (SD = 5.5) years, a mean BMI at baseline of 34.9 (SD = 5.8) kg/m2, and mean waist circumference at baseline of 46.0 cm (SD = 4.9). The sample was predominantly highly educated (86.8% had college or more) and white (61.6%). Full sociodemographic characteristics of the sample are shown in Table 1. No significant differences were observed between usual care and intervention groups with respect to sociodemographic characteristics and baseline BMI and waist circumference values.

### Home neighborhood built environment characteristics

#### Urban design and transit

Among study participants, slightly less than half (48.3%) lived in neighborhoods with high road connectivity ($\ge$ 21 three-way intersections within a 500 m Euclidean buffer, approximately 5–10 min walking distance). Around 45.7% lived in neighborhoods with high availability of transit stops ($\ge$ 21 transit stops within a 1500 m Euclidean buffer, approximately 15–20 min walking time). Meanwhile, more than half (62.9%) lived in areas which had high park density ($\ge$ 1 park within a 1000 m Euclidean buffer, approximately 10–15 min walking time). The average road-network distance to the nearest park from participant’s homes was 2,200 m (approximately 26.5 min walking time) (SD = 2213.9), while the average road-network distance to the nearest transit stop was of 5,211 m (approximately 63 min walking time) (SD = 7228.7). No significant differences in urban design and transit environment characteristics were observed between participants in the intervention and usual care groups at baseline (Table 2).

**Table 2:** Baseline home neighborhood-built environment characteristics among HEALTH study participants (2012–2016)^a^

	Cohort	Usual care	Intervention	*P*-value
	(*n* = 151)	(*n* = 83)	(*n* = 68)	
**Urban Design and Transit Environment, m (SD)**				
Street Connectivity^b^	20.93 (9.26)	20.47 (9.06)	21.49 (9.54)	0.50
Park Density^b^	1.42 (1.46)	1.41 (1.50)	1.44 (1.43)	0.90
Park Distance	2200.31 (2213.92)	2189.72 (2217.25)	2213 (2226.25)	0.94
Metrobus Density^b^	47.68 (61.15)	48.92 (6.90)	46.16 (59.39)	0.78
All-transit Stop Density^b^	47.87 (61.44)	49.12 (63.20)	46.33 (59.66)	0.78
Metrolink Density^b^	0.07 (0.37)	0.07 (0.34)	0.07 (0.40)	0.98
Transit Stop Distance	5211.91 (7228.68)	5237.83 (7485.21)	5180.27 (6957.91)	0.96
**Food Environment, m (SD)**				
Grocery Store Density^b^	1.46 (2.07)	1.64 (2.31)	1.24 (1.72)	0.23
Grocery Store Distance	1836.69 (1891.58)	1891.89 (2138.53)	1769.30 (1550.84)	0.69
Convenience Store Density^b^	0.98 (1.17)	1.05 (1.22)	0.90 (1.12)	0.43
Convenience Store Distance	1795.45 (1784.56)	1828.51 (1837.81)	1755.09 (1645.71)	0.80
Fast Food Density^b^	7.78 (10.26)	9.07 (11.90)	6.21 (7.59)	0.09
Fast Food Distance	1273.26 (1305.38)	1340.46 (1489.70)	1191.24 (1042.36)	0.49

^a^Based on final analytic sample (*n* = 151)

^b^Denisty variables based on participant centric buffers of varying size and types (Euclidean [EB] and network [NB]). Buffer sizes and types per built environment characteristic were defined based on a sensitivity analysis (refer to Additional file 1 online appendix). Street connectivity (500 EB); park, Metrolink, grocery store, and convenience store (1000 EB); Metrobus and all-transit (1500 EB); fast food (1500NB). Density variables dichotomized at the median count of each variable within the determined buffer size and type into high/low. Park, Metrolink, grocery store, convenience store (1); fast food (4); street connectivity, Metrobus, all-transit (21)

^c^Distance variables dichotomized at the median distance in meters and split into high/low distance. Parks (1325.30); transit stops (972.42); grocery stores (1232.12); convenience stores (1344.60); and fast food (865.38)

Note: All distances and buffer radii are measured in meters

#### Food environment

More than half (57%) of the study participants lived in neighborhoods with high grocery store density ($\ge$ 1 store within a 1000 m Euclidean buffer, approximately 10–15 min walking time). Around 56% lived in neighborhoods with high convenience store density ($\ge$ 1 store within a 1000 m Euclidean buffer, approximately 10–15 min walking time), and a little under half (47.6%) lived in neighborhoods with high fast food density ($\ge$ 4 stores within a 1500 m network buffer, approximately 18 min walking time). The average distance to the nearest grocery store was 1,836 m (approximately 22 min walking time) (SD = 1891.6). The average distance to the nearest convenience store was 1,795 m (approximately 22 min walking time) (SD = 1748.6), while the average distance to the nearest fast food restaurant was 1,273 m (approximately 15 min walking time) (SD = 1305.4). Between usual care and intervention groups, no significant differences were found for any of the food environment characteristics.

### Effect modification of the impact of the HEALTH intervention on weight reduction outcomes by neighborhood environment characteristics

Our test for interactions revealed potential effect measure modification of the HEALTH intervention on one or both outcomes of interest (BMI, waist circumference) by all neighborhood environment characteristics examined (Table 3). Therefore, we proceeded to determine the effect of the HEALTH intervention (relative to the usual care arm) across different strata of neighborhood environment features. Population density-adjusted models did not meaningfully change the results when compared to unadjusted models (Additional file 1. Online Appendix). However, there was evidence of confounding by income level for some of the models. Therefore, results are presented for both unadjusted and income-adjusted models (Table 4).

**Table 3:** Testing interactive effect of home neighborhood-built environment characteristics and study arm on weight outcomes.^ab^

	**BMI**		**Waist Circumference**	
	LRT statistic^c^	*P*-value	LRT statistic	*P*-value
**Urban Design and Transit Environment**				
Street Connectivity^d^	1.9	0.17	3.0	**0.08**
Park Density^d^	2.6	**0.11**	3.0	**0.08**
Park Distance^e^	2.3	**0.13**	4.5	**0.03**
Metrobus Density^d^	1.9	0.17	4.8	**0.03**
All-transit Density^d^	1.9	0.17	4.8	**0.03**
Metrolink Density^d^	3.6	**0.06**	4.3	**0.04**
Transit Stop Distance^e^	2.0	0.16	4.6	**0.03**
**Food Environment**				
Grocery Store Density^d^	2.6	**0.11**	3.1	**0.08**
Grocery Store Distance^e^	4.8	**0.03**	7.1	**0.01**
Convenience Store Density^d^	4.1	**0.04**	5.4	**0.02**
Convenience Store Distance^e^	6.0	**0.01**	6.7	**0.01**
Fast Food Density^d^	1.8	0.18	3.2	**0.07**
Fast Food Distance^e^	2.2	**0.14**	2.6	**0.11**

^a^Body mass index and waist circumference

^b^Results show interactive effects from baseline to 24-month follow-up (HEALTH study, 2012–2016)

^c^Likelihood Ratio Test (LRT) statistic follows a Chi-Squared distribution

^d^Density Variables based on participant centric buffers of varying size and types (Euclidean [EB] and network [NB]). Buffer sizes and types per built environment characteristic were defined based on a sensitivity analysis (refer to Additional file 1 online appendix). Street connectivity (500 EB); park, Metrolink, grocery store, and convenience store (1000 EB); Metrobus and all-transit (1500 EB); fast food (1500NB)

^e^Variables measured in meters

Note: Boldface indicates *p* < 0.15 for Likelihood Ratio Test for interaction

Note: based on final analytic sample (*n* = 151)

**Table 4:** Effect modification^a^ of HEALTH intervention on changes to weight outcomes^b^ by home neighborhood-built environment characteristics^c^

	BMI unadjusted models			BMI models adjusted for income			Waist circumference unadjusted models			Waist circumference models adjusted for income		
	beta	SE	*P*-value	beta	SE	*P*-value	beta	SE	*P*-value	beta	SE	*P*-value
**Urban Design and Transit**												
Street Connectivity												
High	N/A	N/A	N/A	N/A	N/A	N/A	-1.73	0.88	0.05	-1.73	0.90	0.06
Low	N/A	N/A	N/A	N/A	N/A	N/A	-2.83	0.88	** < 0.01**	-2.70	0.92	** < 0.01**
Park Density^d^												
High	-2.08	0.61	** < 0.01**	-1.95	0.64	** < 0.01**	-2.73	0.77	** < 0.01**	-2.68	0.79	** < 0.01**
Low	-1.18	0.80	0.14	-1.20	0.85	0.16	-1.68	1.02	0.10	-1.68	1.07	0.12
Park Distance^e^												
High	-1.55	0.66	**0.02**	-1.54	0.70	**0.03**	-1.47	0.84	0.08	-1.57	0.88	0.08
Low	-2.23	0.71	** < 0.01**	-2.09	0.74	** < 0.01**	-3.33	0.90	** < 0.01**	-3.17	0.92	** < 0.01**
Metrobus Density^d^												
High	N/A	N/A	N/A	N/A	N/A	N/A	-1.25	0.90	0.17	-1.06	0.91	0.25
Low	N/A	N/A	N/A	N/A	N/A	N/A	-3.24	0.83	** < 0.01**	-3.40	0.87	** < 0.01**
All-transit Density^d^												
High	N/A	N/A	N/A	N/A	N/A	N/A	-1.25	0.90	0.17	-1.06	0.91	0.25
Low	N/A	N/A	N/A	N/A	N/A	N/A	-3.24	0.83	** < 0.01**	-3.40	0.87	** < 0.01**
Metrolink Density^d^												
High	-1.43	2.32	0.54	-1.29	2.37	0.59	-3.99	2.93	0.18	-4.04	2.95	0.17
Low	-1.75	0.50	** < 0.01**	-1.69	0.52	** < 0.01**	-2.27	0.63	** < 0.01**	-2.24	0.65	** < 0.01**
Transit Stop Distance^e^												
High	N/A	N/A	N/A	N/A	N/A	N/A	-3.20	0.83	** < 0.01**	-3.20	0.86	** < 0.01**
Low	N/A	N/A	N/A	N/A	N/A	N/A	-1.33	0.89	0.14	-1.27	0.91	0.17
**Food Environment**												
Grocery Store Density^d^												
High	-1.41	0.65	**0.03**	-1.23	0.69	0.08	-1.87	0.82	**0.02**	-1.54	0.86	0.08
Low	-2.25	0.75	** < 0.01**	-2.31	0.77	** < 0.01**	-2.98	0.95	** < 0.01**	-3.31	0.96	** < 0.01**
Grocery Store Distance^e^												
High	-2.56	0.67	** < 0.01**	-2.59	0.71	** < 0.01**	-3.59	0.84	** < 0.01**	-3.67	0.87	** < 0.01**
Low	-0.86	0.71	0.23	-0.69	0.74	0.35	-0.87	0.88	0.33	-0.82	0.91	0.37
Convenience Store Density^e^												
High	-1.08	0.65	0.10	-0.97	0.70	0.17	-1.36	0.81	0.10	-1.35	0.86	0.12
Low	-2.58	0.73	** < 0.01**	-2.50	0.75	** < 0.01**	-3.53	0.91	** < 0.01**	-3.35	0.93	** < 0.01**
Convenience Store Distance^e^												
High	-2.73	0.68	** < 0.01**	-2.65	0.69	** < 0.01**	-3.62	0.85	** < 0.01**	-3.45	0.86	** < 0.01**
Low	-0.72	0.69	0.30	-0.58	0.74	0.43	-1.03	0.87	0.24	-1.04	0.92	0.26
Fast Food Density^d^												
High	N/A	N/A	N/A	N/A	N/A	N/A	-1.71	0.90	0.06	-1.37	0.91	0.13
Low	N/A	N/A	N/A	N/A	N/A	N/A	-2.93	0.85	** < 0.01**	-3.22	0.87	** < 0.01**
Fast Food Distance^e^												
High	-2.02	0.67	** < 0.01**	-1.86	0.71	**0.01**	-2.63	0.85	** < 0.01**	-2.63	0.88	** < 0.01**
Low	-1.43	0.73	0.05	-1.51	0.75	0.05	-1.95	0.92	**0.04**	-2.01	0.94	**0.03**

^a^Effect modification was only explored for built environment characteristics which had a *p* < 0.15 for the test for interaction of the given built environment characteristic X study arm. Non-explored built environment X study arm combinations are denoted as N/A (non-applicable)

^b^Body mass index and waist circumference

^c^Results show changes in weight outcomes from baseline to 24-month follow-up (HEALTH study, 2012–2016)

^d^Density variables based on participant centric buffers of varying size and types (Euclidean [EB] and network [NB]). Buffer sizes and types per built environment characteristic were defined based on a sensitivity analysis (refer to online appendix). Street connectivity (500 EB); park, Metrolink, grocery store, and convenience store (1000 EB); Metrobus and all-transit (1500 EB); fast food (1500NB). Density variables dichotomized at the median count of each variable within the determined buffer size and type into high/low. Park, Metrolink, grocery store, convenience store (1); fast food (4); street connectivity, Metrobus, all-transit (21)

^e^Distance variables dichotomized at the median distance in meters and split into great/close distance. Parks (1325.30); transit stops (972.42); grocery stores (1232.12); convenience stores (1344.60); and fast food (865.38)

Note: Variables measured in meters

Note: Boldface indicates statistical significance (*p* < 0.05)

Note: Based on final analytic sample (*n* = 151)

#### Modification by urban design and transit neighborhood environment characteristics

Table 4 shows the stratum specific estimates of the effect of the HEALTH intervention across strata of urban design and transit environmental neighborhood characteristics.

We found *Road-network connectivity (*number of three-way intersections in the home neighborhood, representing *walkability),* moderated the effect of the intervention on the outcome of waist circumference. The intervention was only effective in achieving significant reductions in waist circumference among participants living in neighborhoods with low connectivity (*p* < 0.01), but not for those residing in highly connected neighborhoods (*p* = 0.06).

In terms of *access to parks*, both park density and distance to the nearest park were found to modify the effectiveness of the intervention for both outcomes of interest (reductions in BMI and waist circumference). For the case of park density (number of parks in the home neighborhood, representing park *availability*), the intervention was only effective in achieving significant reductions in BMI and waist circumference for women living in areas with high park density (*p* < 0.01). For the case of park distance (close and far distances to the nearest park, representing park *accessibility),* we found the intervention was effective in achieving significant reductions in BMI for participants, regardless of park distance. However, there was a stronger effect for those living closer to the nearest park (beta -2.09, SE 0.74, *p* < 0.01) relative to those living farther to their nearest park (beta -1.54, SE 0.70, *p* = 0.03). On the other hand, the intervention was only effective in achieving significant reductions in waist circumference for participants living closer to the nearest park (*p* < 0.01) compared to participants living farther away from the nearest park (*p* < 0.08).

Our findings examining potential effect modification of the intervention by *access to public transit* revealed that Metrolink density (representing transit *availability*, and as such, the degree to which there are a variety of options within close range), moderated the effect of the intervention on the outcome of BMI. The intervention only achieved significant reductions in BMI for participants living in areas with a low density of Metrolink stops (*p* < 0.01) compared to participants living in areas with a high density of Metrolink stops (*p* = 0.59). Similarly, Metrobus density, all-transit density, and Metrolink density moderated the effect of the intervention on the outcome of waist circumference, such that the intervention only achieved significant reductions in waist circumference for participants living in areas with low density transit characteristics (*p* < 0.01). Along the same lines, for transit stop distance (representing *accessibility),* we found that the intervention only achieved significant reductions in waist circumference for participants living farther away from their nearest transit stop (*p* < 0.01) compared to participants living closer to the nearest transit stop (*p* < 0.17).

#### Modification by food neighborhood environment characteristics

Table 4 shows the stratum-specific estimates of the effect of the intervention across strata of food environmental neighborhood characteristics.

In terms of *access to grocery stores*, we found that grocery store density (representing grocery store *availability,* and as such, the degree to which there are a variety of options within close range*),* moderated the effect of the intervention for both outcomes. Specifically, the intervention only achieved significant reductions in BMI and waist circumference for participants living in areas with low grocery store density (*p* < 0.01). Similarly, distance to the nearest grocery store (representing *accessibility),* the intervention was found to be effective at reducing BMI and waist circumference only for participants who lived far away from their nearest grocery store (*p* < 0.01).

*Access to convenience stores* modified the effect of the intervention on both outcomes. Density of convenience stores (representing *availability,* i.e., variety of options within close range*),* moderated the effect of the intervention, such that the intervention only achieved significant reductions in BMI and waist circumference for participants living in areas with low convenience store density (*p* < 0.01). Similarly, distance to the nearest convenience store (representing *accessibility*) was also significant moderator of the interventions, which was only effective for participants living far away from their nearest convenience store (*p* < 0.01).

In terms of *fast food density* (representing *availability*), the intervention only resulted in significant reductions in waist circumference for participants living in areas with low density of fast food restaurants (*p* < 0.01), compared to participants living in areas with a high density of fast food restaurants (*p* = 0.13). For fast food distance (representing *accessibility),* we found the intervention only achieved significant reductions in BMI, for participants living farther away from the nearest fast food restaurant (*p* < 0.01) compared to participants living closer to the nearest fast food restaurant (*p* = 0.71). Meanwhile, we found the intervention was effective in achieving significant reductions in waist circumference for all participants, regardless of their distance to fast food outlets. However, there was a stronger effect for those living farther away from their nearest fast food restaurant (beta -2.63, SE 0.88, *p* < 0.01) relative to those living closer to their nearest fast food restaurant (beta -2.01, SE 0.94, *p* = 0.03).

## Discussion

This study presents evidence from the HEALTH study showing that the characteristics of residential neighborhood environments can modify the effectiveness of weight-loss interventions for mothers with overweight or obesity. Our results show that while the main effects of the HEALTH intervention on BMI and waist circumference at 24-months were significant and in the intended direction, effective weight loss occurred mostly among women residing in low-density, low-connectivity neighborhoods with low access to food outlets and transit stops, and high access to parks.

One possible explanation for our findings is that weight-loss interventions, like HEALTH, may work best for “resource deprived” areas, where a small “nudge” can go a long way. *Nudge theory*, proposed by behavioral economists, posits that people’s choices are bounded by their context, and through minimal changes to these contexts, peoples behaviors can change [57]. Hence, it is possible that lifestyle interventions, like HEALTH, can have an outsized effects on behavioral outcomes for participants residing in areas with minimal supports for active living. For example, the HEALTH intervention provided participants with goal-setting techniques for gradually increasing their discretionary physical activity, as well as problem solving skills to meet these goals. These types of strategies may have aided participants in low-resourced neighborhoods in overcoming some of these environmental barriers to healthy living. Other investigators examining the potential moderating role of neighborhood environments on the effectiveness of lifestyle interventions have reported similar results. For instance, Kerr et al. found that their weight-loss intervention for adult men and women was most successful in increasing physical activity levels among participants of less walkable neighborhoods [58]. This interpretation is in line with our results, which revealed that the intervention worked the best for mothers residing in areas with low walkability, access to transit, and access to food stores and services. It must be highlighted that effectively mitigating environmental constraints for healthy living is indeed a central aim of the HEALTH study [57, 59].

Another complementary explanation for these findings is that there may be neighborhood typologies which help optimize the effectiveness of lifestyle interventions. It is possible that in its current form, the HEALTH intervention is most effective in *suburban neighborhoods*, and less so in *inner city, urban core neighborhoods*. Although our analysis did not focus on examining whether certain neighborhood environment characteristics clustered in space, our results are supportive of this hypothesis. Our analysis, consisting of a series of single-environment variable models, revealed that the intervention was mostly effective for those residing in areas with low access to grocery stores, low access to restaurants, low access to public transit, low walkability, and high access to parks. These are all known characteristics of a *suburban neighborhood typology*. On the other hand, our results showed that the effectiveness of the intervention was either weaker or non-significant for mothers living in neighborhoods with high access to grocery stores, restaurants, transit, walkability, and low access to parks (all typical of inner city, urban core neighborhoods). Importantly, our results are not explained by differences in socioeconomic status, as this was accounted for in the analysis. Other studies examining the moderating effect of the built environment on lifestyle behavioral interventions have reported consistent findings [45], but we are the first to do so for weight-loss intervention designed for young mothers. For example, King et al. found that age-related declines in mobility and physical function among older adults were significantly less pronounced among those living in suburban type neighborhoods, when compared to those residing in more urban, compact areas [41].

One important element to consider when designing behavioral interventions intended to be implemented in different types of neighborhoods, is the behavioral domain that they intend to modify, and if and how that matches the place-based characteristics of the participants. For example, many physical activity interventions, including the HEALTH intervention, focus on discretionary (leisure-time or recreational) physical activity [60–62], which is more likely to be supported by neighborhood environments with low residential density, traffic, noise, pollution, and high access to green space. These types of features are most commonly found in the suburbs of US cities, where it has been reported that there are safer and more aesthetically pleasing opportunities for recreational physical activity (e.g., leisure walking) than in higher-density urban areas [41, 63–66]. Conversely, previous evidence on the role of compact, walkable neighborhoods with high density of services and transit (i.e., urban neighborhood typologies) on physical activity (one of the behaviors that influences weight management), is mainly observed as being due to increased utilitarian (e.g., walking or cycling for transport) physical activity in these settings [67–70]. Hence, although it may seem counter-intuitive that lifestyle interventions, such as HEALTH, are less effective in “highly walkable” areas, it may in fact be due to the behavioral domains being targeted by the intervention and their match with the places in which it takes place.

In regard to the food environment, it is worth highlighting that our findings with regards to intervention effectiveness in neighborhoods with high access to fast food restaurant and convenience stores are in the expected direction (the intervention was less effective in these settings). However, this was not the case for supermarket access, which other studies have reported as being directly associated with healthy eating and healthy weight outcomes, while in our study, we found that the intervention was less effective among participants residing in neighborhoods with high access to supermarkets, relative to those with lower access [71, 72]. However, although access to food stores and outlets of any type (healthy or unhealthy) is lower in the suburbs than in the inner-city core, especially when operationalizing access based on walking distances, driving distances to access supermarket are still generally short. In the US, most households own at least one motor vehicle and most people drive to purchase groceries [73–75]. As such, distance to supermarkets may not be as much of a barrier to healthy food access in the suburbs. Additionally, home cooking, which has been associated with healthier eating and better weight outcomes, may be more common in suburban type neighborhoods given their lower access to restaurants and fast food [76, 77]. Altogether, these findings appear to support the suburban neighborhood typology hypothesis – i.e., that it is not individual features (like a supermarket or a fast food store) which are moderating the effectiveness of the intervention, but rather, that the intervention seems to be more effective for people residing in suburban type areas, relative to those in the inner core; all whilst adjusting for socioeconomic level and population density.

Although research has shown the known benefits of compact, walkable neighborhoods with access to services and food vending locations on physical activity and healthy eating [32–34, 78, 79], it may be the case that the suburban area typology with low-density, low-connectivity, and low access to food and transit stops is more supportive for interpersonal lifestyle interventions. Since our analysis was restricted to looking at the possible moderating role of individual neighborhood environmental factors, and not in determining their co-occurrence, future work should examine in more depth the role of neighborhood typologies in the context of lifestyle intervention effectiveness. This will require developing standardized, replicable methods for categorizing neighborhoods based on their clustering environmental factors.

### Limitations and strengths

The findings presented must be interpreted in light of the limitations of the study. Secondary data from public data sources were used to build the geospatial variables, and these data were collected with other intentions beyond public health research (city planning and management, etc.), precluding a comprehensive assessment of data completeness and quality. Some relevant environmental measures were not readily available, including measures of crime or safety, which would have enhanced our analysis. While all attempts were made to temporally-match source built environment data to the time of baseline data collection (2012–2013), exact time-matching was not possible in all instances due to variations on available source GIS data. We did, however, achieve time-matching of the GIS data used for this analysis to be within a range of up to 5-years post-baseline (i.e., built environment variables reflect the ground truth between 2012 and 2017). While built environments do change over time (e.g., the number of fast food restaurants in a neighborhood can change over time), it is highly unlikely that any given neighborhood environment would change to a degree such that it would modify the assigned classification across dichotomized built environment measures (e.g., turn a neighborhood classified as having a high number of parks to become one that has a low number of parks, or vice versa). We measured access to built environment characteristics through density and distance-based measures but did not assess the quality of these features, nor participant perceptions of their neighborhood environment. Another possible limitation is that we dichotomized the built environment variables examined as potential moderators. It was decided to do so to allow for easier interpretation of the moderating effects of the built environment on the intervention by a broad public health audience, and, because of the exploratory nature of this analysis. Further, we dichotomized built environment features via a median-split approach. As such, “low” and “high” access categories are defined by the available range of values in the City of St. Louis, and may not be reflective of relative high versus low access to environmental assets in other contexts. The scope of this analysis was limited to using changes in BMI and waist circumference as the primary outcomes of interest; however, future analyses should explore the potential moderating roles of neighborhood environmental features on changes in intermediary behavioral outcomes, such as measures of physical activity and dietary behaviors. Additionally, we did not have an optimal measure of urbanicity available and relied on a measure of population density to approximate this complex construct. Another limitation of the study is the potential introduction of selection bias due to differential losses to follow-up across arms in the parent study. Finally, the scope of this study focused on assessing individual built environment features, and did not analyze the co-occurrence of these features or other environmental factors which influence physical activity and healthy eating (i.e., food prices, social support, crime, neighborhood aesthetics). Future research should be conducted to explore the impact of co-occurring features on study effectiveness as well as the impact of other environmental factors not explored in this analysis.

Our study also had some key strengths which are important to underscore. We included two outcomes for weight status (BMI score and waist circumference). Waist circumference is a more sensitive measure of weight loss and specifically weight loss associated with better health outcomes [80, 81]. Also, we provide a unique contribution to the literature around the role of the built environment on interpersonal interventions for weight loss among overweight or obese young women. Few, if any studies have explored this relationship among women of preschool children with overweight or obesity, limiting our knowledge around how effective interpersonal interventions are in varying built environment contexts for this population.

## Conclusions

This study sheds light with respect to the characteristics of neighborhoods where the HEALTH study intervention is most effective. Specifically, we found the intervention to be most effective for women residing in low-density, low-connectivity neighborhoods, with low access to food outlets and transit stops, and high access to parks (i.e., suggestive of a suburban neighborhood typology). Further steps should be taken to scale up the HEALTH intervention to these types of settings.

Future research should also look toward adapting the intervention to maximize effectiveness in compact neighborhoods with high access to restaurants, grocery stores, with high connectivity, and high access to public transit but low access to parks (most likely to be inner city, urban core neighborhoods). Additional mixed-methods research is required to identify the barriers operating in these settings, which may be limiting intervention effectiveness. This will allow for new iterations of the intervention to become better tailored, allowing to maximize its impact for mothers residing in all types of neighborhoods, thus ensuring it benefits the greatest possible number of people and contributes to reducing health inequities.

## Supplementary Information

**Additional file 1. **

## Data Availability

The datasets generated and/or analyzed during the current study are available from the corresponding author on reasonable request.

## Abbreviations

HEALTH: Healthy Eating and Active Living Taught at Home
PAT: Parents as Teachers
