# Supplementary material for: Does the neighborhood built environment moderate the effectiveness of a weight-loss intervention for mothers with overweight or obesity? Findings from the Healthy Eating and Active Living Taught at Home (HEALTH) study
Source: Int J Behav Nutr Phys Act. 2022 Oct 1;19:130. doi: 10.1186/s12966-022-01368-z (PMC9526987; doi:10.1186/s12966-022-01368-z)
Supplement: Supplementary file 1 — Additional file 1. [file 12966_2022_1368_MOESM1_ESM.docx]

Online appendix

**Does the neighborhood built environment moderate the effectiveness of weight-loss interventions for mothers with overweight or obesity: The Healthy Eating and Active Living Taught at Home (HEALTH) study**

Authors

Amanda S. Gilbert, MSW, MPH^1^

*Deborah Salvo, PhD^1^

Rachel Tabak, PhD, RD

Debra Haire-Joshu**,** PhD, RN, MS, MA

^1^Prevention Research Center in St. Louis, Brown School at Washington University in St. Louis, St. Louis, MO

*Corresponding Author One Brookings Drive Campus Box 1196 St. Louis, MO 63130 a.s.gilbert@wustl.edu

**Table of contents**

Section 1: Sensitivity analysis for identifying the optimal buffer type and size for exploring the moderating effect of neighborhood environment characteristics and HEALTH study outcomes (BMI and waist circumference)…………………………….……………………………………..3

- 1. Introduction……………………….……………………………………………………...……3
  2. Methods ……………………………………………………….........................................…... 3
  3. Results ………………………………………………………………………………………...3

Section 2: Unadjusted and population density adjusted models examining the moderating effect of the built environment on HEALTH study outcomes (BMI and waist circumference)…...……5

- 1. Introduction ………………………………………………………………………………...…5
  2. Methods ………………………………………………………………………………….……5
  3. Results …………………………………………………………………………….…….…….5
     1. BMI models …………………………………………………………………..…………..5
     2. Waist circumference models…….………………………………………………...…..…..7

Section 3: References…………………………………………………………………………….8

**Section 1:** **Sensitivity analysis for identifying the optimal buffer type and size for exploring the moderating effect of neighborhood environment characteristics and HEALTH study outcomes (BMI and waist circumference)**

- 1. **Introduction**

A sensitivity analysis was conducted to determine for each built environment buffer-based measure, which specific buffer (type and size) was most strongly correlated with the HEALTH study outcomes of interest (body mass index and waist circumference). Depending on the spatial environment and population of interest, individual behaviors vary. As such, sensitivity analyses are necessary for identifying the geospatial unit of analyses (buffer size and types) that most meaningfully captures built environment influences on individual behaviors, given a certain geospatial area.

**1.2 Methods**

After geocoding all participant addresses, we built a series of participant centric buffers of varying radii (250, 500, 1000, 1500) in meters (M). Two types of buffers were used, including Euclidian buffers (EB), measured as the crow flies, as well as Network buffers (NB), measured through underlying road networks to find all possible routes from the participant to the end point. (see figure 1). Next, we conducted a sensitivity analysis using Pearson correlation coefficients to determine for each built environment buffer-based measure, which specific buffer (type and size) was most strongly correlated with the HEALTH study outcomes of interest (body mass index and waist circumference).

The final buffer selected for analysis was that with the highest Pearson correlation coefficient for the relation between the given built environment feature and the two HEALTH study outcomes of interest. When the highest correlation coefficient between a given built environment variable and obesity measures was different across outcomes (BMI vs. waist circumference), we selected the buffer with correlations in the same direction and that were in the expected direction for the given built environment to obesity indicator relationship. When no consistent pattern was observed, we selected the buffer with the highest correlation coefficient for waist circumference, since waist circumference is a more sensitive measure of abdominal obesity, which is most detrimental to health.^1–3^

**1.3 Results**

Online Appendix Table 1, below, presents the results of the sensitivity analysis. Bolded Pearson correlation coefficients indicate the buffer type and size used in the final models exploring moderating effects of the built environment on HEALTH study outcomes of BMI and waist circumference.

**Online Appendix Table 1.** Pearson Correlation Coefficients of built environment density variables and weight outcomes^a^ with buffer sizes and types.^b^

|  | **250m NB^c^** | **250m EB^d^** | **500m NB^c^** | **500m EB^d^** | **1000m NB^c^** | **1000m EB^d^** | **1500m**  **NB^c^** | **1500m EB^d^** |
| --- | --- | --- | --- | --- | --- | --- | --- | --- |
| **Grocery Store** |  |  |  |  |  |  |  |  |
| BMI^e^ | 0.0275 | 0.0357 | 0.0498 | 0.0709 | 0.1259 | **0.1303** | 0.1085 | 0.0974 |
| WC^f^ | 0.0512 | 0.0861 | 0.0709 | 0.0779 | 0.1148 | **0.1202** | 0.1207 | 0.1024 |
| **Fast Food** |  |  |  |  |  |  |  |  |
| BMI^e^ | 0.0808 | 0.0601 | 0.1057 | 0.0662 | 0.0970 | 0.1115 | **0.1321** | 0.0617 |
| WC^f^ | 0.0977 | 0.0639 | 0.0666 | 0.0093 | 0.0458 | 0.0497 | **0.0799** | 0.0169 |
| **Convenience Store** |  |  |  |  |  |  |  |  |
| BMI^e^ | -0.0848 | -0.1059 | 0.0163 | 0.0822 | 0.1893 | **0.1934** | 0.1463 | 0.1328 |
| WC^f^ | -0.1181 | -0.1060 | -0.0028 | 0.0298 | 0.1586 | **0.1595** | 0.1294 | 0.1404 |
| **All-transit** |  |  |  |  |  |  |  |  |
| BMI^e^ | 0.0303 | 0.0533 | 0.0645 | 0.0984 | 0.0989 | 0.0979 | 0.0909 | **0.0959** |
| WC^f^ | 0.0806 | 0.0716 | 0.0869 | 0.0893 | 0.0989 | 0.0824 | 0.0904 | **0.1091** |
| **Metrobus Stops** |  |  |  |  |  |  |  |  |
| BMI^e^ | 0.0303 | 0.0530 | 0.0637 | 0.0981 | 0.0992 | 0.0987 | 0.0908 | **0.0965** |
| WC^f^ | 0.0806 | 0.0725 | 0.0863 | 0.0889 | 0.0835 | 0.0916 | 0.0911 | **0.1099** |
| **Metrolink Stops** |  |  |  |  |  |  |  |  |
| BMI^e^ | NA | 0.0347 | 0.0728 | 0.0728 | 0.0146 | **-0.0239** | 0.0516 | 0.0117 |
| WC^f^ | NA | -0.0174 | 0.0757 | 0.0757 | -0.0709 | **-0.0975** | -0.0307 | -0.0048 |
| **Three-way Intersection** |  |  |  |  |  |  |  |  |
| BMI^e^ | -0.0043 | 0.0088 | 0.0415 | **0.0852** | 0.0403 | 0.0223 | 0.0338 | 0.0018 |
| WC^f^ | -0.0438 | -0.0007 | 0.0543 | **0.0603** | 0.0123 | -0.0304 | -0.0071 | -0.0735 |
| **Parks** |  |  |  |  |  |  |  |  |
| BMI^e^ | 0.0068 | -0.0646 | -0.0653 | 0.0174 | 0.0586 | **0.1159** | 0.1069 | 0.0984 |
| WC^f^ | -0.0146 | -0.0064 | 0.0247 | 0.0459 | 0.0599 | **0.1217** | 0.1189 | 0.1006 |

^a^Health study outcomes of Body mass index and waist circumference.

^b^Buffers are participant-centric

^c^NB (Network Buffer)

^d^EB (Euclidean Buffer)

^e^BMI (Body mass index)

^f^WC (waist circumference)

All buffer radii were measured in meters

**Bold** denotes selected buffer type and size

**Section 2: Unadjusted and population density adjusted models examining the moderating effect of the built environment on HEALTH study outcomes**

**(BMI and waist circumference)**

- 1. **Introduction**

We initially explored unadjusted models for the moderating effect of the built environment on HEALTH study outcomes of BMI and waist circumference. The results of this analysis indicated the HEALTH intervention was more or only effective for reducing BMI and waist circumference for participants living in contexts where walkability was lower and there were fewer built environment resources like food outlets or transit stops, but where open areas for recreation were readily available (e.g., parks). Since these built environment characteristics are often found in suburban areas, we ran an analysis adjusting all models for population density to account for the potential confounding effect of urbanicity.

- 1. **Methods**

For the final models in which “built environment x experimental arm” significant interactions were identified, we used outcome measures (BMI, waist circumference) at 24-months as the dependent variable while only adjusting for the baseline outcome measure.^30,31^ We report these as unadjusted models in appendix section 2.3, since they did not control for anything other than the baseline outcome measure. We then ran all models adjusted for population density. Population density was estimated using census block group total population size data and intersecting it with each participant's buffer. Through spatial apportionment, the slices of each census block group intersecting the buffer were used to compute a weighted average, to estimate total population size for each participant-centric buffer. This number was divided by the buffer area to estimate population density per buffer and expressed as total population per Kilometer squared.

- 1. **Results**
     1. **BMI models**

Results of the population density-adjusted models are shown in Online Appendix Table 2, below. Population density adjusted models for the moderating effect of the built environment on BMI did not meaningfully change the results when compared to unadjusted models. Because of this, the final results included in the main tables of the paper are not adjusted for population density.

**Online Appendix Table 2.** Outcomes of interactions explored for HEALTH participants home neighborhood-built environment characteristics and changes in BMI.^ab^

|  | **Unadjusted Models** | | |  | **Models adjusted for density** | | |
| --- | --- | --- | --- | --- | --- | --- | --- |
|  | beta | SE | P-value |  | beta | SE | P-value |
| **Urban Design and Transit Environment** |  |  |  |  |  |  |  |
| Park Density^c^ |  |  |  |  |  |  |  |
| High | -2.08 | 0.61 | **<0.01** |  | -2.06 | 0.62 | **<0.01** |
| Low | -1.18 | 0.80 | 0.14 |  | -1.17 | 0.80 | 0.15 |
| Park Distance^d^ |  |  |  |  |  |  |  |
| High | -1.55 | 0.66 | **0.02** |  | -1.55 | 0.67 | **0.02** |
| Low | -2.23 | 0.71 | **<0.01** |  | -2.24 | 0.71 | **<0.01** |
| Metrolink Density^c^ |  |  |  |  |  |  |  |
| High | -1.43 | 2.32 | 0.54 |  | -1.55 | 2.35 | 0.51 |
| Low | -1.75 | 0.50 | **<0.01** |  | -1.78 | 0.51 | **<0.01** |
| **Food Environment** |  |  |  |  |  |  |  |
| Grocery Store Density^c^ |  |  |  |  |  |  |  |
| High | -1.41 | 0.65 | **0.03** |  | -1.42 | 0.66 | 0.03 |
| Low | -2.25 | 0.75 | **<0.01** |  | -2.25 | 0.75 | **<0.01** |
| Grocery Store Distance^d^ |  |  |  |  |  |  |  |
| High | -2.56 | 0.67 | **<0.01** |  | -2.59 | 0.67 | **<0.01** |
| Low | -0.86 | 0.71 | 0.23 |  | -0.92 | 0.72 | 0.20 |
| Convenience Store Density^c^ |  |  |  |  |  |  |  |
| High | -1.08 | 0.65 | 0.10 |  | -1.08 | 0.67 | 0.11 |
| Low | -2.58 | 0.73 | **<0.01** |  | -2.58 | 0.74 | **<0.01** |
| Convenience Store Distance^d^ |  |  |  |  |  |  |  |
| High | -2.73 | 0.68 | **<0.01** |  | -2.73 | 0.68 | **<0.01** |
| Low | -0.72 | 0.69 | 0.30 |  | -0.77 | 0.71 | 0.28 |
| Fast Food Distance^d^ |  |  |  |  |  |  |  |
| High | -2.02 | 0.67 | **<0.01** |  | -2.02 | 0.68 | **<0.01** |
| Low | -1.43 | 0.73 | 0.05 |  | -1.51 | 0.74 | **0.04** |
| ^a^Body mass index  ^b^Results show changes changes in weight outcomes from baseline to 24-month follow-up (HEALTH study, 2012-2016)  ^c^Density variables based on participant centric buffers of varying size and types (Euclidean [EB] and network [NB]). Buffer sizes and types per built environment characteristic were defined based on a sensitivity analysis (refer to online appendix). Street connectivity (500 EB); park, Metrolink, grocery store, and convenience store (1000 EB); Metrobus and all-transit (1500EB); fast food (1500NB). Density variables dichotomized at the median count of each variable within the determined buffer size and type into high/low. Park, Metrolink, grocery store, convenience store (1); fast food (4); street connectivity, Metrobus, all-transit (21). | | | | | | | |
| ^d^Distance variables dichotomized at the median distance in meters and split into great/close distance. Parks (1325.30); transit stops (972.42); grocery stores (1232.12); convenience stores (1344.60); and fast food (865.38).  Note: Used 1500 meter network buffer for population density when adjusting for distance based variables. Selection based on a buffer size and type that is inclusive of all median values of distance variables and representative of distance traveled using the road networks.  Note: Variables measured in meters.  Note: **Boldface** indicates statistical significance (p <. 0.05). | | | | | | | |

- - 1. **Waist circumference models**

Results of the population density-adjusted models are shown in Online Appendix Table 3, below. Population density adjusted models for the moderating effect of the built environment on waist circumference did not meaningfully change the results when compared to unadjusted models. Because of this, the final results included in the main tables of the paper are not adjusted for population density.

**Online Appendix Table 3.** Effect modification^a^ of HEALTH intervention on changes in waist circumference, by home neighborhood-built environment characteristics.^b^

|  | **Unadjusted models** | | |  | **Models adjusted for density** | | |
| --- | --- | --- | --- | --- | --- | --- | --- |
|  | beta | SE | P-value |  | beta | SE | P-value |
| **Urban Design and Transit Environment** |  |  |  |  |  |  |  |
| Street Connectivity |  |  |  |  |  |  |  |
| High | -1.73 | 0.88 | 0.05 |  | -1.74 | 0.89 | 0.05 |
| Low | -2.83 | 0.88 | **<0.01** |  | -2.93 | 0.90 | **<0.01** |
| Park Density^c^ |  |  |  |  |  |  |  |
| High | -2.73 | 0.77 | **<0.01** |  | -2.70 | 0.79 | **<0.01** |
| Low | -1.68 | 1.02 | 0.10 |  | -1.65 | 1.02 | 0.11 |
| Park Distance^d^ |  |  |  |  |  |  |  |
| High | -1.47 | 0.84 | 0.08 |  | -1.50 | 0.85 | 0.08 |
| Low | -3.33 | 0.90 | **<0.01** |  | -3.38 | 0.91 | **<0.01** |
| Metrobus Density^c^ |  |  |  |  |  |  |  |
| High | -1.25 | 0.90 | 0.17 |  | -1.23 | 0.92 | 0.18 |
| Low | -3.24 | 0.83 | **<0.01** |  | -3.23 | 0.84 | **<0.01** |
| All-transit Density^c^ |  |  |  |  |  |  |  |
| High | -1.25 | 0.90 | 0.17 |  | -1.23 | 0.92 | 0.18 |
| Low | -3.24 | 0.83 | **<0.01** |  | -3.23 | 0.84 | **<0.01** |
| Metrolink Density^c^ |  |  |  |  |  |  |  |
| High | -3.99 | 2.93 | 0.18 |  | -3.92 | 2.97 | 0.19 |
| Low | -2.27 | 0.63 | **<0.01** |  | -2.25 | 0.64 | **<0.01** |
| Transit Stop Distance^d^ |  |  |  |  |  |  |  |
| High | -3.20 | 0.83 | **<0.01** |  | -3.19 | 0.83 | **<0.01** |
| Low | -1.33 | 0.89 | 0.14 |  | -1.29 | 0.91 | 0.16 |
| **Food Environment** |  |  |  |  |  |  |  |
| Grocery Store Density^c^ |  |  |  |  |  |  |  |
| High | -1.87 | 0.82 | **0.02** |  | -1.84 | 0.83 | 0.03 |
| Low | -2.98 | 0.95 | **<0.01** |  | -2.94 | 0.95 | **<0.01** |
| Grocery Store Distance^d^ |  |  |  |  |  |  |  |
| High | -3.59 | 0.84 | **<0.01** |  | -3.63 | 0.84 | **<0.01** |
| Low | -0.87 | 0.88 | 0.33 |  | -0.93 | 0.90 | 0.30 |
| Convenience Store Density^c^ |  |  |  |  |  |  |  |
| High | -1.36 | 0.81 | 0.10 |  | -1.33 | 0.83 | 0.11 |
| Low | -3.53 | 0.91 | **<0.01** |  | -3.52 | 0.92 | **<0.01** |
| Convenience Store Distance^d^ |  |  |  |  |  |  |  |
| High | -3.62 | 0.85 | **<0.01** |  | -3.62 | 0.85 | **<0.01** |
| Low | -1.03 | 0.87 | 0.24 |  | -1.07 | 0.89 | 0.23 |
| Fast Food Density^c^ |  |  |  |  |  |  |  |
| High | -1.71 | 0.90 | 0.06 |  | -1.75 | 0.92 | 0.06 |
| Low | -2.93 | 0.85 | **<0.01** |  | -2.94 | 0.85 | **<0.01** |
| Fast Food Distance^d^ |  |  |  |  |  |  |  |
| High | -2.63 | 0.85 | **<0.01** |  | -2.64 | 0.85 | **<0.01** |
| Low | -1.95 | 0.92 | **0.04** |  | -2.02 | 0.94 | **0.03** |
| ^a^ Effect modification was only explored for built envrionment characteristics which had a p<0.15 for the test for interaction of the given built environment characteristic X study arm. Non-explored built environment X study arm combinations are denoted as N/A (non-applicable)  ^b^Results show changes in weight outcomes from baseline to 24-month follow-up (HEALTH study, 2012-2016)  ^c^ Density variables based on participant centric buffers of varying size and types (Euclidean [EB] and network [NB]). Buffer sizes and types per built environment characteristic were defined based on a sensitivity analysis (refer to online appendix). Street connectivity (500 EB); park, Metrolink, grocery store, and convenience store (1000 EB); Metrobus and all-transit (1500EB); fast food (1500NB). Density variables dichotomized at the median count of each variable within the determined buffer size and type into high/low. Park, Metrolink, grocery store, convenience store (1); fast food (4); street connectivity, Metrobus, all-transit (21).  ^d^Distance variables dichotomized at the median distance in meters and split into great/close distance. Parks (1325.30); transit stops (972.42); grocery stores (1232.12); convenience stores (1344.60); and fast food (865.38).  Note: Variables measured in meters. | | | | | | | |
| Note: **Boldface** indicates statistical significance (p < 0.05). | | | | | | | |

**Section 3: References**

1. Jacobs EJ, Newton CC, Wang Y, et al. Waist circumference and all-cause mortality in a large US cohort. *Arch Intern Med*. 2010;170(15):1293-1301. doi:10.1001/archinternmed.2010.201

2. Lee CMY, Huxley RR, Wildman RP, Woodward M. Indices of abdominal obesity are better discriminators of cardiovascular risk factors than BMI: a meta-analysis. *J Clin Epidemiol*. 2008;61(7):646-653. doi:10.1016/j.jclinepi.2007.08.012

3. Janssen I, Katzmarzyk PT, Ross R. Waist circumference and not body mass index explains obesity-related health risk. *Am J Clin Nutr*. 2004;79(3):379-384. doi:10.1093/ajcn/79.3.379
